# Exploring Pain Phenotyping in Cervicogenic Headache Management

**DOI:** 10.1007/s11916-025-01420-0

**Published:** 2025-12-13

**Authors:** Michael Cropes, Albojay Deacon, Evan O Nelson, Daniel Deuel, Andrew Sandgren, Alaa Abd-Elsayed, Tiffany Houdek

**Affiliations:** 1 Department of Family Medicine and Community Health, School of Medicine and Public Health, Doctor of Physical Therapy Program, University of Wisconsin-Madison, Madison, WI USA; 2 Department of Physical Therapy, College of Health Sciences, Marquette University, Milwaukee, WI USA; 3 University of Wisconsin Hospitals and Clinics, Madison, WI USA; 4 University of Wisconsin-Madison, Madison, WI USA

**Keywords:** Rehabilitation, Pain mechanisms, Pain phenotyping, Quantitative sensory testing

## Abstract

**Purpose of Review:**

Intervention trials for cervicogenic headache (CGH) often yield equivocal results with marked treatment effect heterogeneity, possibly reflecting variations in underlying pain mechanisms throughout the CGH population. Pain phenotyping or classifying patients into subgroups based on their predominant pain mechanism may facilitate more precise CGH treatment. This review aims to explore the role of pain phenotyping in CGH management.

**Recent Findings:**

Clinical evidence suggests two predominant pain mechanisms in the CGH population: nociceptive and nociplastic. Arguably, treatments for nociceptive pain should address the source of peripheral nociception, and treatments for nociplastic pain should address factors contributing to maladaptive central pain modulation. Due to centrally mediated analgesic effects, muscle relaxants are strongly recommended for managing both nociceptive and nociplastic CGH pain. Antidepressant medications may be most relevant for nociplastic CGH pain. Cervical spinal mobilization and manipulation interventions are strongly recommended for both nociceptive and nociplastic CGH pain. Nociplastic CGH pain may benefit from educational interventions regarding lifestyle factors such as physical activity, diet and weight management, sleep hygiene, and stress reduction. The anesthetic blockade, glucocorticoid injection, and radiofrequency denervation are strongly recommended for nociceptive CGH pain. Patients with persistent nociplastic CGH pain may benefit from neuromodulation interventions.

**Summary:**

Pain phenotyping may facilitate more precise clinical management of patients with CGH. This review provides evidence-informed recommendations for CGH pain phenotyping, including specific subgroups, clinical criteria, and stratified treatment approaches. Further prospective investigation is needed to determine the effects of pain phenotyping on clinical outcomes in patients with CGH.

## Introduction

Cervicogenic headache (CGH) is classified as a secondary headache, attributed to nociceptive sources within the cervical spine [1]. Symptoms are likely mediated by both peripheral and central neurophysiological mechanisms [2]. The estimated prevalence of CGH in the general population is between 0.2% and 4.1% [3–5]. Individuals with CGH report lower health-related quality of life than the general population and lower physical functioning than individuals with migraine or tension-type headache [6].

Variable treatment effectiveness has been demonstrated for numerous CGH interventions including therapeutic exercise and manual therapy [7–9], dry needling [10], interventional pain procedures [2, 11, 12], and surgical procedures [13, 14]. In a 2024 meta-analysis of CGH treatments, interventions such as dry needling, therapeutic exercise, spinal joint thrust manipulation, soft tissue techniques, and muscle energy techniques were associated with meaningful improvements in short-term headache characteristics [15]. However, interpretation of the article was obscured by the presence of considerable treatment effect heterogeneity (I^2^ >70%) across the studied interventions, a finding often observed in clinical trials for patients with painful conditions [15, 16]. While the observed treatment effect heterogeneity may have been influenced by use of inconsistent treatment parameters, it may have also reflected truly heterogeneous patient responses to the same intervention, with the presence of variable underlying pain mechanisms throughout the study population serving as an important interaction factor [17].

Through innovations in pain management, we may better understand and address confounding factors such as treatment effect heterogeneity, ultimately allowing us to deliver the “right treatment, in the right dose, to the right patient, at the right time” [16, 17]. With a growing understanding of the neurophysiological mechanisms of pain, contemporaries have called for further clinical investigation into pain phenotyping, or classifying patients based on their pain experiences, underlying mechanisms, and responses to dedicated interventions [16, 18]. Pain phenotypes may be established based on physiological measures, observable clinical features, and clinical examination. Quantitative sensory testing (QST) is one clinical examination method used to inform patient phenotyping [19, 20]. QST comprises a set of standardized psychophysical assessments that quantify multimodal somatosensory functions of both large-diameter (Aβ-fibers; group II) and small-diameter (Aδ- and C-fibers; groups III and IV) sensory nerve fibers, revealing signs of functional loss (hypoesthesia, hypoalgesia) or gain (hyperesthesia, hyperalgesia, allodynia) that may elucidate the peripheral and central mechanisms contributing to a patient’s pain experience [21–25]. QST has been used to identify unique phenotypic subgroups in patients diagnosed with neuropathic pain [26], knee osteoarthritis [27], low back pain [28], and irritable bowel syndrome [29]. At least one prospective study has demonstrated the value of QST-informed phenotyping for effectively stratifying pharmacologic treatment in patients with peripheral neuropathic pain [30].

QST has yielded heterogeneous patterns of somatosensory functions when administered to CGH study participants, suggesting the presence of non-uniform pain mechanisms throughout the CGH population [31–35]. Presumably, patients with painful conditions will respond best to interventions that most effectively target their predominant pain mechanism(s) [36]. Accordingly, for any given clinical population, the identification of subgroups with shared underlying pain mechanisms, as evidenced by similar phenotypic profiles, may facilitate more effective treatment stratification. Given the potential value of pain phenotyping to improve clinical outcomes in the CGH population, future investigation is needed to (1) identify pain mechanisms-based CGH phenotypes and (2) evaluate the effectiveness of phenotype-stratified CGH treatment. This narrative review aims to explore the role of pain phenotyping in CGH management, based on existing evidence regarding CGH diagnosis, pathophysiology, and treatment.

## Diagnosis

CGH diagnosis is based on a detailed history and clinical examination, albeit no validated reference standard exists [37]. A key feature of CGH diagnosis is suspected involvement of the cervical spine as a primary nociceptive origin, which may be evaluated via physical examination alone or in combination with anesthetic blocks [2]. CGH diagnosis can be complicated by its overlapping symptomatology with other headache disorders such as migraine [2, 38]. Compared to migraine, the findings of limited cervical range of motion, pain provocation with neck movement, shoulder and arm discomfort, and posterior onset of head pain may be more common in patients with CGH [39]. Table 1 summarizes CGH diagnostic criteria from the 3rd edition of the International Classification for Headache Disorders (ICHD-3) [1] and the Cervicogenic Headache International Study Group (CHISG) [40].

**Table 1:** Diagnostic Classification Systems for Cervicogenic Headache

ICHD-3 Diagnostic Criteria	CHISG Major Diagnostic Criteria
I. Any headache fulfilling criterion [III]	I. Symptoms and signs of neck involvement:
II. Clinical and/or imaging evidence of a disorder or lesion within the cervical spine or soft tissues of the neck, known to be able to cause headache	i. precipitation of head pain, similar to the usually occurring one:
III. Evidence of causation demonstrated by at least two of the following:	a. by neck movement and/or sustained awkward head positioning, and/or:
i. headache has developed in temporal relation to the onset of the cervical disorder or appearance of the lesion	b. by external pressure over the upper cervical or occipital region on the symptomatic side
ii. headache has significantly improved or resolved in parallel with improvement in or resolution of the cervical disorder or lesion	ii. restriction of the range of motion in the neck
iii. cervical range of motion is reduced, and headache is made significantly worse by provocative maneuvers	iii. ipsilateral neck, shoulder, or arm pain of a rather vague nonradicular nature or, occasionally, arm pain of a radicular nature.
iv. headache is abolished following diagnostic blockade of a cervical structure or its nerve supply	II. Confirmatory evidence by diagnostic anesthetic blockades
IV. Not better accounted for by another ICHD-3 diagnosis	III. Unilaterality of the head pain, without side shift

*ICHD-3 *3rd edition of the International Classification for Headache Disorders, *CHISG* Cervicogenic Headache International Study Group,

## Pathophysiology

Contemporary research suggests three mechanisms-based categories of pain: nociceptive, neuropathic, and nociplastic [41, 42]. Nociceptive pain arises from actual or threatened damage to non-neural tissues and is due to the activation of peripheral nociceptors. Neuropathic pain is caused by damage or disease affecting the somatosensory nervous system. Nociplastic pain arises from altered nociception despite no clear evidence of tissue damage. Physiologically, nociceptive pain is associated with peripheral sensitization and hyperexcitability of first-order nociceptive neurons, manifesting as localized pain and primary hyperalgesia (increased pain response to a noxious stimulus that normally provokes pain) at the site of suspected nociceptor activation. Both nociplastic and neuropathic types of pain are associated with hyperexcitability of central nervous system (CNS) neurons and maladaptive central modulation of pain, manifesting as more diffuse pain, secondary hyperalgesia (increased pain responses within undamaged tissues), allodynia (pain response to a stimulus that does not normally provoke pain), and temporal summation of pain (progressively stronger pain response to successive noxious stimuli of the same intensity). Notably, peripheral sensitization may play a role in initiating, maintaining, and modulating CNS neuronal hyperexcitability in cases of nociplastic and neuropathic pain [43].

Symptom presentations throughout the CGH population are likely attributable to nociceptive and/or nociplastic pain mechanisms. The nociceptive structures implicated in CGH include the upper cervical spinal joints, ligaments, muscles, dura, and arteries, all of which are innervated by C1-C3 spinal nerve afferents [39, 44]. CGH symptoms may be experienced throughout the craniocervical distribution of C1-C3 as well as the craniofacial distribution of the trigeminal nerve (cranial nerve Ⅴ). These patterns of symptom distribution are likely facilitated by the convergence of first-order nociceptive afferent neurons from distinct head/neck regions on the same second-order neuron in the trigeminocervical nucleus, the continuous longitudinal column of gray matter between the trigeminal nucleus caudalis and the dorsal horns of the upper cervical spinal cord [45–47]. Study participants with CGH have demonstrated increased serum levels of nitric oxide and pro-inflammatory cytokines (interleukin-1𝛃 and tumor necrosis factor-ɑ) [48], substances that can sensitize both peripheral and central nociceptive neurons.

A 2011 study supported the role of centrally mediated pain mechanisms in CGH [49]. Researchers administered a QST battery to seventeen participants with chronic CGH (CGH group) and ten participants with chronic neck pain without CGH (non-CGH group). Compared to the non-CGH group, the CGH group demonstrated diffuse mechanical hyperalgesia of multiple cranial and cervical muscles on the symptomatic side of the body, a finding indicative of central neuronal hyperexcitability [50]. The CGH group also demonstrated hypersensitivity to both cold and warm stimuli on the symptomatic side of the face, further suggesting the presence of facilitatory central mechanisms, particularly involving the trigeminocervical nucleus. In a separate study of the CGH population, eighteen participants’ QST results demonstrated a trend towards diffuse mechanical hyperalgesia across cephalic and extra-cephalic muscle testing sites; however, the finding was only present in 50% of the sample, suggesting centrally-mediated pain mechanisms may exhibit nonuniform contributions in patients with CGH [31].

## Pain Phenotyping

The aforementioned evidence suggests a patient’s CGH pain presentation may be influenced by nociceptive and/or nociplastic mechanisms. In cases of predominantly nociceptive CGH pain, symptoms are attributed to peripheral nociceptive input. In cases of predominantly nociplastic CGH pain, symptoms are likely initiated by a peripheral nociceptive input but maintained by various drivers of central neuronal hyperexcitability [31, 43]. For patients with nociplastic CGH pain, interventions solely targeting a peripheral source of nociception (e.g. facet joint injections) may lead to insufficient clinical outcomes [36, 50]. To enhance treatment selection accuracy, researchers have suggested phenotyping (or subgrouping) patients based on shared findings (e.g., symptom characteristics, QST results, or psychosocial factors) that may reflect shared underlying pain mechanisms [31]. Specific criteria have been published to guide pain mechanisms-based phenotyping in the chronic low back pain population [51]; however, no such criteria have been published for the CGH population. Considering the absence of available evidence specific to CGH, Table 2 proposes clinical criteria for two CGH pain phenotypes, nociceptive and nociplastic, informed by pain phenotyping literature regarding other clinical populations [51, 52]. Notably, a neuropathic CGH pain phenotype is not proposed in Table 2, as such a clinical presentation would be more consistent with a diagnosis of trigeminal neuralgia, occipital neuralgia, or upper cervical radiculopathy. Notably, future research is needed to determine the reliability and validity of the criteria proposed in Table 2.

**Table 2:** Proposed Clinical Criteria for Pain Phenotyping in Cervicogenic Headache

	**Nociceptive CGH Pain Phenotype**	**Nociplastic CGH Pain Phenotype**
Causes	Pain arising from activation of peripheral nociceptors within injured head and/or neck tissues innervated by C1-C3 or CN V	Pain arising from altered nociception despite no clear evidence of tissue damage
Related Physiological Phenomena	Peripheral sensitization, immune-mediated inflammation, peripheral neurogenic inflammation, central convergence-projection	Central sensitization, heterosynaptic facilitation, central neurogenic inflammation, maladaptive descending modulation of pain
Clinical Findings	Pain location is discrete, with possible referred pain throughout sensory distribution of spinal nerves C1-C3 and CN V Evoked pain response is consistent with suspected severity of tissue trauma Primary hyperalgesia observed with noxious stimulation of injured head and/or neck tissues Positive symptom response to anesthetic blockade (e.g., upper cervical joint injection, medial branch block)	Pain location is regional, multifocal, or widespread Possible spontaneous pain Evoked pain responses are unexpectedly disproportionate to somatosensory inputs Secondary hyperalgesia observed with noxious stimulation of non-injured tissues Possible allodynia Decreased conditioned pain modulation efficiency Associated fatigue, sleep disturbance, cognitive disturbances, anxiety, and/or depressed mood

cervicogenic headache, CHG; cranial nerve V or trigeminal nerve, CN V

## Nociceptive CGH Pain Phenotype

 Nociceptive CGH pain arises from actual or threatened tissue injury and associated peripheral nociceptor activation within non-neural head and/or neck tissues innervated by spinal nerves C1-C3 or the trigeminal nerve (cranial nerve V). Underlying physiological phenomena associated with nociceptive pain include peripheral sensitization, immune-mediated inflammation, and peripheral neurogenic inflammation [53, 54]. Nociceptive CGH pain distribution is localized to the site of peripheral nociceptor activation, albeit referred pain may be observed throughout the craniocervical and craniofacial regions, possibly mediated by the central convergence-projection phenomenon [45, 51]. When noxious somatosensory input is applied to the injured tissue (e.g., via deep palpation or sustained postures), primary hyperalgesia is observed, and the evoked pain response is consistent with the suspected severity of tissue trauma. Given the dominant role of peripheral nociceptor activation in nociceptive CGH pain, anesthetic blockades are expected to provide symptom relief.

## Nociplastic CGH Pain Phenotype

 Nociplastic CGH pain arises from altered nociceptive processing despite no clear evidence of associated tissue injury [42]. Underlying physiological phenomena associated with nociplastic pain include central sensitization, heterosynaptic facilitation, central neurogenic inflammation, and maladaptive descending modulation of pain [55–57]. Nociplastic CGH pain distribution may be regional, multifocal, or widespread, and patients may experience spontaneous pain [58]. When various (potentially multimodal) somatosensory stimuli are applied to the patient, the evoked pain responses are unexpectedly disproportionate to the degree of input. Secondary hyperalgesia may be observed when noxious stimuli are applied to non-injured tissues of the head, neck, or distant sites [59]. Allodynia, a painful response to a stimulus that does not normally provoke pain (e.g., mechanical brushing, light touch), may also be observed in the head, neck, or distant sites. Conditioned pain modulation testing protocols, which examine the human presentation of diffuse noxious inhibitory control, may demonstrate inefficient descending pain modulation [55, 60, 61]. Nociplastic pain conditions may be associated with fatigue, sleep disturbance, cognitive disturbances, anxiety, and/or depressed mood [55].

##  Pain Phenotype-stratified Treatment

 Using a pain phenotype-stratified treatment approach, clinicians aim to select interventions that optimally target a patient’s predominant pain phenotype and associated physiological mechanisms. The potential value of pain phenotyping to enhance treatment selection appears promising [51, 55, 62]. Treatments for nociceptive pain address sources of peripheral nociception, treatments for neuropathic pain address neuropathology, and treatments for nociplastic pain address factors contributing to maladaptive central pain modulation [25, 55, 62]. Some treatment modalities have the potential to address multiple pain phenotypes. For example, in 38 study participants with chronic knee osteoarthritis, a passive tibiofemoral joint mobilization intervention was associated with improvements in primary mechanical hyperalgesia [63] (nociceptive pain) as well as improvements in both secondary mechanical hyperalgesia [63] and conditioned pain modulation [64] (nociplastic pain).

 Currently, there is a lack of evidence to guide the implementation of pain phenotype-stratified treatment in the CGH population. In 2021, The Chinese Association for the Study of Pain published a guideline for CGH management, including a list of twenty-four recommendations graded “weak” or “strong” based on quality of evidence, balance between desirable and undesirable effects, values, preferences, and costs [65]. Notably, the concept of pain phenotyping was not considered in the guideline but has the potential to enhance its clinical value. Table 3 proposes how various “strong” recommendations from the 2021 guideline may be integrated into a pain phenotype-stratified treatment approach.

**Table 3:** Proposed Pain Phenotype-stratified Treatment of Cervicogenic Headache

	**Nociceptive CGH Pain Phenotype**	**Nociplastic CGH Pain Phenotype**
Pharmacologic	Muscle relaxants	Muscle relaxants Antidepressants for patients with severe anxiety or depression
Non-pharmacologic	Cervical spinal joint non-thrust mobilization and thrust manipulation Patient education	Cervical spinal joint non-thrust mobilization and thrust manipulation Patient education Psychological assessment and intervention for patients with refractory severe symptoms
Procedural	Anesthetic blockade Glucocorticoid injection Radiofrequency denervation for patients with intractable symptoms	Neuromodulation for patients with intractable symptoms
Surgical	Not recommended without compelling evidence of a surgically amenable lesion that is refractory to all reasonable nonsurgical treatments	

Adapted from Xiao H, Peng BG, Ma K, et al. Expert panel’s guideline on cervicogenic headache: The Chinese Association for the Study of Pain recommendation. World J Clin Cases. 2021;9(9):2027-2036. doi:10.12998/wjcc.v9.i9.2027

## Pharmacologic Interventions

 The 2021 guideline strongly recommends pharmacologic treatment as a first-line therapy for CGH [65]. The use of muscle relaxants receives a strong recommendation, with authors highlighting the centrally mediated analgesic effects and potential benefits in both acute symptom management and prevention [65]. Considering the central analgesic actions of muscle relaxants, these medications may be relevant to the management of both nociceptive and nociplastic CGH pain phenotypes. The guideline also strongly recommends antidepressants for patients with severe anxiety and depression [65]. Given the common finding of mood problems across nociplastic conditions [55], antidepressants may be more relevant for patients with nociplastic CGH pain.

## Non-Pharmacologic Interventions

 The guideline strongly recommends cervical spinal joint mobilization and manipulation manual interventions [65]. Operationally defined, non-thrust mobilization procedures involve repeated low-velocity joint movements of varying amplitudes, while joint thrust manipulation procedures involve a single high-velocity, low-amplitude thrust directed at a target joint and often resulting in an audible cavitation [66]. In a scoping review of manual therapy procedures using animal models, spinal thrust manipulation was shown to elicit changes in muscle spindle activity, central neuronal activity, multifidus electromyographic response, and systemic immunologic response [67]. This preclinical evidence suggests joint-biased manual therapy interventions directed at the cervical spine may be indicated for both nociceptive and nociplastic CGH pain phenotypes.

 Additionally, the guideline strongly recommends educational interventions addressing pain neurophysiology, posture, and neck-specific exercises [65]. Nijs et al suggest pain education interventions should focus on the presumed source of nociception for patients with nociceptive pain, and should explain central sensitization for patients with nociplastic pain [62]. Furthermore, Fitzcharles et al suggest patients with nociplastic pain may benefit from educational interventions regarding lifestyle factors such as physical activity, diet and weight management, sleep hygiene, and stress reduction [55].

 Finally, the guideline makes a strong recommendation that patients with refractory severe CGH need psychological assessment and intervention [65], consistent with broader treatment recommendations for patients with nociplastic pain conditions [55].

 Arguably, physical therapists are well positioned to deliver and/or refer patients to other healthcare professionals for the non-pharmacologic interventions discussed in this section, considering physical therapists’ training in addition to the relatively long duration and frequent nature of physical therapy visits.

## Procedural Interventions

 The guideline strongly recommends use of anesthetic joint injections or nerve blocks, and intra-articular glucocorticoid injections in patients with CGH [65]. Radiofrequency denervation and neuromodulation interventions are conditionally recommended for patients with intractable symptoms [65]. Neuromodulation techniques for headache management include pulsed radiofrequency [68], peripheral nerve stimulation, and spinal cord stimulation [69], and may produce analgesia through spinal and supraspinal mechanisms [70]. Procedural interventions that target a peripheral nociceptive driver (e.g., anesthetic blockade, glucocorticoid injection, radiofrequency denervation) may be most appropriate for patients with predominant CGH nociceptive pain. Neuromodulation techniques, given their suspected central analgesic actions, may be appropriate for patients with nociplastic CGH pain.

## Surgical Interventions

 The guideline strongly recommends against surgery for patients with CGH, unless there is compelling evidence of a surgically amenable lesion causing the condition that is refractory to all reasonable nonsurgical interventions [65]. The authors suggest surgery may be beneficial for CGH etiologies such as atlantoaxial joint osteoarthritis and upper cervical intervertebral disc pathology. Accordingly, surgical intervention may be conditionally indicated for patients with predominant nociceptive CGH pain.

## Clinical Application

 The following section offers a framework for applying pain phenotyping to the clinical management of patients with CGH. Once a diagnosis of CGH has been established using ICHD-3 [1] or CHISG criteria [40] (Table 1), the clinician gathers additional information to determine whether the patient’s predominant CGH pain phenotype is nociceptive or nociplastic (Table 2). The clinician uses the interview and body diagram [71] to assess the patient’s pain characteristics. A discrete pain location suggests a nociceptive phenotype. A regional, multifocal, or widespread distribution of pain and/or spontaneous nature of pain suggests a nociplastic phenotype. The clinician also uses the interview and relevant standardized measures to screen for fatigue, sleep dysfunction, cognitive disturbances, anxiety, or depressed mood. The presence of these comorbidities may indicate a nociplastic pain phenotype. Throughout the examination process, the clinician evaluates the relationship between somatosensory stimulus and evoked pain response. When the evoked pain response is unpredictable or disproportionate to the stimulus, such as severe pain provocation associated with gentle neck movement or brief maintenance of a head posture, nociplastic pain is suspected. During the physical examination the clinician performs static (monofilament) or dynamic (brush) mechanical allodynia testing on the head, neck, trunk, and extremities. The presence of allodynia in any of these regions suggests a nociplastic pain phenotype. Next, the clinician administers potentially-noxious examination procedures to both local (e.g., neck) and distant (e.g., extremity) tissues: range of motion, passive accessory intervertebral motion assessment, and deep palpation or pressure pain threshold (PPT) testing via pressure algometry [72]. Hypersensitive responses upon noxious stimulation of suspected injured head or neck tissues reflect primary hyperalgesia and nociceptive pain. Hypersensitive responses to noxious stimulation of non-injured tissues throughout the head, neck, or distant body regions reflect secondary hyperalgesia and nociplastic pain. Finally, conditioned pain modulation testing protocols [60] may be administered to measure the patient’s efficiency of endogenous pain modulation, with a finding of decreased efficiency suggesting a nociplastic pain phenotype.

 The following two case scenarios illustrate the clinical evaluation and treatment of patients with differing CGH phenotypes.

## Case Scenario #1: Nociceptive CGH Pain

Patient 1 is a middle-aged adult with two months of episodic right-sided headache radiating from the suboccipital region toward the forehead. Pain is aggravated with neck movement, particularly after prolonged occupational deskwork. Physical examination is remarkable for a restricted range of motion and provocation of localized suboccipital pain with right rotation cervical range of motion. The clinician observes hypersensitivity to palpation of the right suboccipital muscles and bilateral upper trapezii, interpreted as primary and secondary hyperalgesia, respectively. Passive accessory intervertebral motion at the C2-C3 level elicits pain in the right forehead region (note, while referred pain may be mediated by a central phenomenon, it does not necessarily indicate a nociplastic pain phenotype). Although the patient demonstrates one clinical sign consistent with nociplastic pain (secondary hyperalgesia with palpation of bilateral upper trapezii), the preponderance of findings leads the clinician to classify the patient with predominantly nociceptive CGH pain attributable to activation of peripheral nociceptors within the upper cervical spinal joints and muscles. The phenotype-informed multidisciplinary treatment plan includes muscle relaxants for acute symptom management, patient education regarding the presumed triggers of nociception and related ergonomic considerations, and neck-specific exercises and manual therapies to target both peripheral and central analgesic mechanisms. The patient regularly follows up with a care team member such as a physical therapist to monitor progress and adjust the treatment plan as needed. Lack of patient improvement indicates referral to an interventional pain management provider.

## Case Scenario #2: Nociplastic CGH Pain

Patient 2 is a middle-aged adult with six months of worsening right-sided headache radiating from the suboccipital region toward the forehead. Upon additional screening, the patient endorses a recent onset of neck and upper back pain, difficulty with sleep initiation, low mood, and impaired concentration at work (note, patients may not offer this information without solicitation, and the use of standardized screening forms can facilitate a more comprehensive understanding of patient history). The patient’s physical examination is remarkable for positive dynamic (brush) mechanical allodynia testing in the posterior neck region. The patient demonstrates reduced range of motion and provocation of diffuse upper back, neck, and head pain with cervical range of motion into extension and bilateral rotation. Primary hyperalgesia is observed with passive accessory intervertebral motion at the C2-C3 level. Secondary hyperalgesia is observed with palpation of the right upper trapezius, infraspinatus, and brachioradialis muscles. Generally, there is an unexpectedly disproportionate relationship between the somatosensory stimuli being delivered to the patient and the evoked pain responses. The clinician classifies the patient with predominantly nociplastic CGH pain. In this scenario, phenotype-informed management may still include interventions targeting potential sources of peripheral nociception (e.g., anesthetic upper cervical joint injections); however, interventions primarily target central nociceptive processing and pain modulation. Muscle relaxants are prescribed for centrally mediated analgesic effects. Care team members explain the role of the central nervous system in nociceptive processing, educate the patient on sleep hygiene principles, and highlight the benefits of routine exercise for managing pain, mood, and cognitive functions. Neck-specific exercises, general exercises, and manual therapies are administered to target both peripheral and central analgesic mechanisms. Lack of patient improvement indicates referrals to mental health and/or interventional pain management providers.

## Conclusion

CGH is a painful condition associated with physical disability and reduced quality of life. The results of existing CGH clinical trials have been difficult to interpret due to significant variability in patient outcomes, likely influenced by varying degrees of underlying peripheral and central pain mechanisms throughout the CGH population. The effectiveness of CGH management may be enhanced through the implementation of pain phenotype-informed treatment selection. This review proposes evidence-informed pain phenotyping clinical criteria and stratified treatment considerations for the CGH population. Future research is needed to investigate the prognostic and treatment-modifying effects of pain phenotyping in CGH management.

## Key References

- Edwards RR, Schreiber KL, Dworkin RH, et al. Optimizing and Accelerating the Development of Precision Pain Treatments for Chronic Pain: IMMPACT Review and Recommendations. J Pain. 2023;24(2):204-225. doi:10.1016/j.jpain.2022.08.010.
  - This narrative review highlights the presence of common pathophysiologic mechanisms and shared phenotypes across chronic pain syndromes and supports the implementation of pain phenotyping to advance precision pain medicine.
- Mingels S, Granitzer M, Schmid AB, Dankaerts W. Individual endogenous pain modulation profiles within a multidimensional context of people with cervicogenic headache – A retrospective exploratory study. Musculoskelet Sci Pract. 2023;67: None. doi:10.1016/j.msksp.2023.102855.
  - The results of this retrospective study suggest the presence of heterogeneous pathophysiological pain mechanisms and pain phenotypes throughout the cervicogenic headache population.
- Nijs J, Kosek E, Chiarotto A, et al. Nociceptive, neuropathic, or nociplastic low back pain? The low back pain phenotyping (BACPAP) consortium’s international and multidisciplinary consensus recommendations. Lancet Rheumatol. 2024;6(3): e178-e188. doi:10.1016/S2665-9913(23)00324-7. 
  - There is a paucity of literature to guide pain phenotyping in the cervicogenic headache population. This paper provides consensus recommendations defining clinical criteria of three unique pain phenotypes in the chronic low back pain population. The recommendations provided in this paper were used as a framework to guide pain phenotyping in the cervicogenic headache population.

## Data Availability

No datasets were generated or analysed during the current study.
